# Assessment of Pancreatic Exocrine Insufficiency in Patients with Dyspepsia: Clinical Utility of the PEI-Test in Identifying and Monitoring Response to Enzyme Replacement Therapy

**DOI:** 10.3390/jcm15062297

**Published:** 2026-03-17

**Authors:** Ahmet Said Dundar, Kadir Demir, Mehmet Bayram, Eda Nur Duran, Hafize Uzun, Omur Tabak

**Affiliations:** 1 Department of Internal Medicine, Kanuni Sultan Suleyman Training and Research Hospital, University of Health Sciences, Istanbul 34303, Türkiye; ahmetsaiddundar@gmail.com (A.S.D.); enurduran@gmail.com (E.N.D.); 2 Division of Gastroenterohepatology, Department of Internal Medicine, Istanbul Faculty of Medicine, Istanbul University, Istanbul 34403, Türkiye; kadirdmr@yahoo.com; 3 Department of Gastroenterology, Türkiye Hospital, Istanbul 34381, Türkiye; drmhbayram@gmail.com; 4 Department of Medical Biochemistry, Faculty of Medicine, Istanbul Atlas University, Istanbul 34403, Türkiye; huzun59@hotmail.com

**Keywords:** pancreas exocrine insufficiency, dyspepsia, PEI test, fecal steatocrit, patient-reported outcome

## Abstract

**Background and Objectives:** Functional dyspepsia (FD) often overlaps with Pancreatic Exocrine Insufficiency (PEI), leading to diagnostic delays. We aimed to evaluate the prevalence of PEI in patients presenting dyspeptic symptoms using the survey-based PEI test and to assess the clinical response to Pancreatic Enzyme Replacement Therapy (PERT). **Methods:** This study included 91 patients with PEI and 58 control subjects. PEI was evaluated using the PEI Patient-Reported Outcome (PRO) instrument and classified as mild, moderate, or severe according to the 18-item PEI test. Objective fat malabsorption was assessed by the acid steatocrit method using a gravimetric assay. Patients diagnosed with PEI received PERT, and treatment response was evaluated at follow-up with a repeat PEI test. **Results:** When the case and control groups were compared in terms of PEI scores, a statistically significant difference was found (*p* < 0.001). Fecal steatocrit value was found to be statistically significant with the PEI score (*p* = 0.017). No statistically significant difference was found between amylase, lipase, vitamin D, vitamin B12, and folic acid and the PEI score (*p* = 0.789, *p* = 0.299, *p* = 0.865, *p* = 0.153, and *p* = 0.855, respectively). A statistically significant difference was found between the pre-treatment PEI score and the post-treatment PEI score (*p* < 0.001). The mean pre-treatment PEI score was 1.52 ± 0.50, while the post-treatment PEI score was 0.42 ± 0.48. Approximately 72% reduction in PEI score was observed with treatment. **Conclusions:** The PEI test may represent a useful, non-invasive tool for identifying suspected pancreatic dysfunction in patients initially diagnosed with functional dyspepsia. Early integration of this tool into clinical practice can improve symptom control and prevent the misclassification of PEI as a purely functional disorder.

## 1. Introduction

Dyspepsia is a clinical syndrome characterized by a broad spectrum of upper gastrointestinal symptoms, including epigastric pain, bloating, early satiety, and nausea. These symptoms, whether meal-related or independent, can present as acute or chronic conditions. Dyspepsia can be categorized as uninvestigated, organic, or functional, depending on the presence or absence of identifiable pathology on endoscopic evaluation. In the absence of alarm symptoms, patients with uninvestigated dyspepsia can be managed empirically following a clinical evaluation [1].

Approximately 20–25% of patients presenting with dyspepsia have underlying organic causes. However, in 75–80% of cases, no underlying organic cause can be found. This group is defined as functional dyspepsia [2]. The etiology of organic dyspepsia includes peptic ulcer, Helicobacter pylori (HP) infection, gastroesophageal reflux disease, biliary pain, chronic abdominal pain, gastric and esophageal cancer, gastroparesis, pancreatitis, carbohydrate malabsorption, medications, infiltrative diseases of the stomach (Crohn’s disease, sarcoidosis), metabolic disorders, systemic disorders, intestinal parasites, abdominal cancers (especially pancreatic cancer) [3].

Pancreatic Exocrine Insufficiency (PEI) is defined as a reduction in pancreatic enzyme activity in the intestinal lumen that is insufficient for normal digestion [4]. While fecal elastase-1 (FE-1) is commonly used to assess pancreatic capacity, its diagnostic sensitivity is notably low in mild cases of PEI [5,6]. Consequently, objective measures of fat malabsorption, such as the acid steatocrit, remain clinically valuable. Acid steatocrit is a gravimetric method that provides a rapid and quantitative assessment of fecal fat excretion. It has been validated as a reliable screening tool for steatorrhea, showing a high correlation with the 72 h quantitative fecal fat analysis the historical gold standard while being significantly more practical for routine clinical use [7]. Unlike FE-1, which measures enzyme concentration, the acid steatocrit directly reflects the physiological consequence of enzyme deficiency: impaired lipid digestion. Its sensitivity in detecting significant malabsorption has been reported at approximately 80–90%, making it a robust marker for identifying patients who are likely to benefit from Pancreatic Enzyme Replacement Therapy (PERT) [6,7].

By integrating the symptom-based PEI-test with the acid steatocrit, we aimed to overcome the limitations of single-marker diagnostics in a primary care setting. We hypothesized that a significant proportion of patients categorized with “uninvestigated” or “functional” dyspepsia possess underlying PEI that remains undetected by conventional structural investigations. We proposed that the PEI test scores would significantly correlate with fecal steatocrit levels and that targeted treatment would result in a substantial reduction in dyspeptic symptom scores, confirming the test’s predictive value for treatment success. The primary objective of this study was to evaluate the diagnostic utility of the PEI test in identifying PEI among patients presenting with dyspeptic symptoms. Furthermore, we aimed to determine the correlation between diagnostic scores and clinical response to enzyme replacement therapy, assessing the test’s efficacy in clinical decision-making.

## 2. Materials and Methods

### 2.1. Ethical Considerations

The study protocol was approved by the Academic Ethics Committee of University of Health Sciences, Istanbul Kanuni Sultan Suleyman Training and Research Hospital (Approval No: 92, 26 July 2022).

### 2.2. Study Design and Population

This clinical retrospective study was conducted at the Departments of Gastroenterology and Internal Medicine of the University of Health Sciences, Istanbul Kanuni Sultan Suleyman Training and Research Hospital. Data was retrospectively analyzed for patients who presented dyspeptic complaints between 2019 and 2022. Data for both the patient and control groups were retrospectively retrieved from the hospital information management system through a comprehensive review of medical records.

#### 2.2.1. Inclusion Criteria

To ensure a rigorous differential diagnosis of dyspeptic symptoms, the study included only patients who had undergone a comprehensive diagnostic work-up. This evaluation comprised a complete blood count (CBC), a standard biochemistry profile, and serum levels of amylase and lipase. Additionally, all participants were screened for secondary causes of dyspepsia through stool microscopy, fecal Helicobacter pylori antigen testing, and transabdominal ultrasonography. Screening for Celiac disease was performed by measuring serum Immunoglobulin A (IgA) levels and anti-tissue transglutaminase (tTG) IgA or anti-endomysium (EMA) IgA antibodies. Furthermore, upper gastrointestinal endoscopy (gastroscopy) was mandatory for inclusion in the study group to exclude structural pathologies.

#### 2.2.2. Exclusion Criteria

To maintain a homogenous study group and isolate Pancreatic Exocrine Insufficiency (PEI) as the primary focus, the following patients were excluded from the study:(i) Patients diagnosed with intestinal parasitosis, *H. pylori* infection, Celiac disease, or Inflammatory Bowel Disease (IBD). Celiac disease was excluded because it is a well-recognized cause of secondary pancreatic exocrine insufficiency, which could confound the relationship between dyspeptic symptoms and primary pancreatic dysfunction. (ii) Those with evidence of cholelithiasis. (iii) Individuals with a history of gastric or intestinal surgical interventions. (iv) Pregnant or lactating women. (v) Cases lacking a follow-up PEI test.

#### 2.2.3. Study Population and Flow

**Figure 1:**
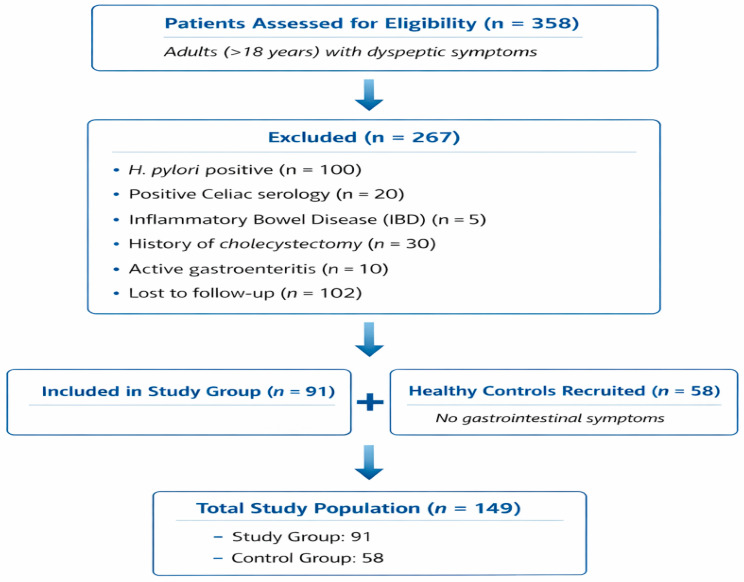
Participant flow diagram of the study population.

A total of 358 adults presenting with dyspeptic symptoms to the Gastroenterology and Internal Medicine outpatient clinics were initially assessed for eligibility. After applying exclusion criteria, 267 individuals were excluded due to *H. pylori* positivity (*n* = 100), positive celiac serology (*n* = 20), inflammatory bowel disease (*n* = 5), history of cholecystectomy (*n* = 30), active gastroenteritis (*n* = 10), or loss to follow-up (*n* = 102). Consequently, 91 eligible patients were included in the study group. Additionally, 58 asymptomatic healthy individuals were recruited as controls. The final study population comprised 149 participants (91 in the study group and 58 in the control group) (Figure 1).

#### 2.2.4. Control Group Selection

The control group consisted of 59 individuals who presented to the outpatient clinic for routine health check-ups and had no history of gastrointestinal complaints. To ensure comparability, the control subjects were selected from a similar age and gender distribution as the case group. Exclusion criteria for the control group were identical to those applied to the study cohort, specifically excluding individuals with a history of gastrointestinal surgery, systemic disorders, metabolic diseases, or any active infection. Furthermore, subjects in the control group were confirmed to have no dyspeptic symptoms through a detailed clinical anamnesis.

### 2.3. Diagnostic Evaluation

To facilitate differential diagnosis of dyspeptic symptoms, only patients who had undergone a comprehensive clinical work-up were included. This evaluation encompassed:

***Laboratory Analysis:*** Complete blood count (CBC), a standard biochemistry panel, serum amylase, and lipase levels.

***Gastrointestinal Screening:*** Stool microscopy, fecal *Helicobacter pylori* antigen testing, and upper gastrointestinal endoscopy (gastroscopy).

***Imaging:*** Transabdominal ultrasonography.

***Celiac Disease Screening:*** Serum Immunoglobulin A (IgA) levels, along with anti-tissue transglutaminase (tTG) IgA and/or anti-endomysium (EMA) IgA titers.

#### 2.3.1. Data Collection

Patient records, including demographic data, laboratory results (amylase, lipase, vitamin levels), and fecal steatocrit values, were retrieved from the hospital’s electronic automation system.

#### 2.3.2. Diagnostic Tool

Patients were diagnosed with Pancreatic Exocrine Insufficiency (PEI) based on the survey-based PEI-test scoring system proposed by Johnson et al. [8]. To ensure clinical accuracy, only tests administered and evaluated by a physician were included; patient-reported or self-administered tests were excluded from the study. For the treatment cohort, patients identified as having PEI were initiated on Pancreatic Enzyme Replacement Therapy (PERT). Only those who underwent a follow-up (post-treatment) PEI test to evaluate clinical response were included in the final analysis.

#### 2.3.3. PEI-Test Scoring and Evaluation

The PEI test is an 18-item survey designed to assess clinical symptoms across three primary domains: abdominal symptoms (**Part A**), bowel movement-related symptoms (**Part B**), and the impact on quality of life for diagnosed patients (**Part C**). Symptom severity over the preceding seven days was evaluated using a 5-point Likert scale (0: Never to 4: Always).

The scoring methodology distinguishes between newly diagnosed and follow-up patients to ensure clinical relevance. Specific formulas were applied to calculate the total PEI score, which then categorized the severity of pancreatic exocrine insufficiency into mild, moderate, or severe clinical states. The comprehensive structure of the survey, the mathematical calculation of scores, and the corresponding severity classifications are detailed in Table 1.

#### 2.3.4. Fecal Fat Excretion

Fecal fat excretion was assessed using the acid steatocrit method. Spot stool samples were analyzed via a gravimetric assay to determine the steatocrit value, providing a quantitative measure of fat malabsorption [8]. This technique was employed to evaluate the correlation between PEI scores and objective markers of maldigestion [9]. FE-1 testing is widely used for the diagnosis of pancreatic exocrine insufficiency; however, it was not available for all patients in our center during the study period. Therefore, acid steatocrit was used as an objective indicator of fat malabsorption. Steatocrit analysis was performed in the clinical biochemistry laboratory according to standard laboratory procedures; however, duplicate measurements were not systematically performed due to the retrospective design of the study.

### 2.4. Statistical Analysis

Statistical analyses were performed using SPSS software (version 22.0 for Windows; IBM Corporation, Chicago, IL, USA). Descriptive statistics were presented as numbers and percentages for categorical variables, while continuous (numerical) variables were expressed as mean ± standard deviation (SD). The normality of the data distribution was assessed using the Shapiro–Wilk test. For the comparison of categorical data between groups, the Chi-square test was employed. In the comparison of continuous variables between two groups, Student’s *t*-test was used for normally distributed parameters, whereas the Mann–Whitney U test was applied for non-normally distributed data. For comparisons involving more than two groups, one-way ANOVA was utilized for normally distributed variables, and the Kruskal–Wallis test was used for non-normally distributed parameters. Correlation analyses were conducted using Pearson’s correlation for normally distributed variables and Spearman’s rank correlation for non-normally distributed variables. A *p*-value of <0.05 was considered statistically significant for all analyses.

## 3. Results

### Demographic Characteristics

The demographic distribution of the study and control groups is summarized in Table 2. The mean age of the study group was 42 ± 16 years (range: 18–79), while the mean age of the control group was 47 ± 17 years (range: 18–72). No statistically significant difference was observed between the two groups regarding age (*p* = 0.240). In terms of gender distribution, the study group consisted of 19 males (20.9%) and 72 females (79.1%). The control group included 19 males (32.8%) and 39 females (67.2%). Statistical analysis revealed no significant difference in gender composition between the groups (*p* = 0.105), ensuring that the cohorts were well-matched for demographic variables.

When the case and control groups were compared in terms of the PEI score, a statistically significant difference was found (*p* = 0.000). The fecal steatocrit value was found to be statistically significant with the PEI score (*p* = 0.017).

When the PEI score was compared with amylase, lipase, HbA1c, vitamin D, vitamin B12, and folic acid, no statistically significant difference was found (*p* = 0.580, *p* = 0.185, *p* = 0.374, *p* = 0.834, *p* = 0.388, and *p* = 0.169, respectively).

A moderate positive correlation was found between the fecal steatocrit value and the PEI score (r = 0.50). The correlation was found to be statistically significant (*p* < 0.001).

No correlation was found between amylase, lipase, vitamin D, vitamin B12, or folic acid and the PEI score (r =0.022, r = −0.086, r =0.014, r = −0.015, r = −0.118, respectively). The correlation was not statistically significant (*p* = 0.789, *p* = 0.299, *p* = 0.865, *p* = 0.153, and *p* = 0.855, respectively).

A statistically significant difference was found between the pre-treatment PEI score and the post-treatment PEI score (*p* < 0.001).

A weak negative correlation was found between the pre-treatment PEI score and the post-treatment PEI score (r = −0.184). However, this correlation was not found to be statistically significant (*p* = 0.086).

**Figure 2:**
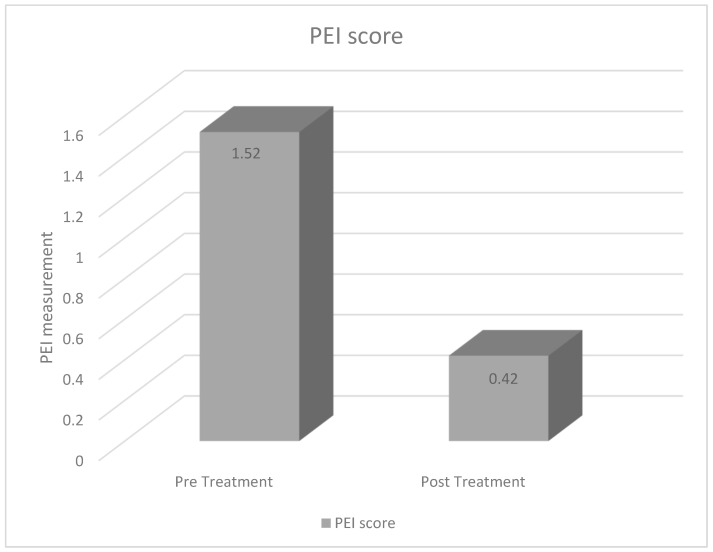
Comparison of PEI scores before and after treatment.

The pre-treatment PEI score meant: While the PEI score was 1.52 ± 0.50, the PEI score was determined to be 0.42 ± 0.48 after treatment. A reduction of approximately 72% in PEI score was observed after treatment, as shown in Figure 2.

## 4. Discussion

The most important finding of this study is that the PEI score was significantly higher in patients compared with healthy controls and that fecal steatocrit emerged as the only parameter significantly associated with PEI. A statistically significant difference in PEI scores was observed between the case and control groups. Moreover, fecal steatocrit showed both a significant association with PEI and a moderate positive correlation, indicating that increased intestinal fat loss is closely linked to PEI severity. In contrast, no significant associations were identified between PEI score and serum amylase, lipase, HbA1c, vitamin D, vitamin B12, or folic acid levels, suggesting that routine biochemical markers may have limited value in reflecting PEI-related clinical burden in this population. Importantly, treatment resulted in a marked clinical improvement, with the mean PEI score decreasing from 1.52 ± 0.50 to 0.42 ± 0.48, corresponding to an approximate 72% reduction. Together, these findings demonstrate the substantial therapeutic responsiveness of the condition and validate the PEI score by showing its strong association with fecal steatocrit as an objective reference of fat malabsorption.

Dyspepsia accounts for a significant proportion of outpatient admissions worldwide, although prevalence rates vary between 16% and 66% due to differing diagnostic criteria and ethnic backgrounds [10,11,12]. Despite its high prevalence, approximately 75–80% of patients presenting with dyspeptic symptoms have no identifiable organic cause and are subsequently classified as having functional dyspepsia (FD) [13]. Various pathophysiological mechanisms have been proposed for FD, including gastrointestinal motility disorders, visceral hypersensitivity, immune and mucosal dysfunction, alterations in the gut microbiome, and abnormal interactions within the brain–gut axis [14].

The present study focused on patients presenting with dyspepsia in internal medicine and gastroenterology clinics where no organic pathology was detected through standard investigations. Our primary objective was to evaluate the diagnostic and follow-up utility of the PEI test in patients whose symptoms suggested underlying PEI. The demographic profile of our cohort (mean age 43.68 years) was highly consistent with the studies by Johnson et al., who reported mean ages of 36 and 43.3 years in their respective PEI validation cohorts [8,15]. Notably, our study had a higher female predominance (79.1%) compared to Johnson’s groups (45.7–42%). This female majority in our study may reflect a higher rate of healthcare-seeking behavior among women for dyspeptic complaints in our regional population, leading to a more frequent diagnosis of functional symptoms.

PEI is characterized by impaired digestion and malabsorption resulting from deficient pancreatic secretion. While chronic pancreatitis is the leading cause in adults, and cystic fibrosis in children, other etiologies include pancreatic tumors and gastrointestinal surgeries [16]. Historically, PEI diagnosis has been challenging because classic symptoms like steatorrhea and weight loss often appear only in advanced stages [17]. In earlier stages, patients frequently present with non-specific symptoms such as abdominal pain, bloating, and nausea, which overlap significantly with FD [18]. Because the symptoms of dyspepsia and pancreatic exocrine insufficiency overlap considerably, symptom-based assessments should be interpreted with caution. Tests such as fecal elastase, pancreatic function tests, and radiolabeled triglyceride breath test can be helpful in diagnosing PEI [19]. However, there is no accepted gold standard test, and in clinical practice, patients are diagnosed based on their symptoms. Response to treatment is often assessed by symptom severity; there is no routinely used test that assesses treatment response. For this reason, and because the symptoms of PEI patients are subjective, a tool like the PEI test is gaining value in clinical practice. Our study hypothesized that a PEI-test validated against fecal elastase could provide a reliable tool for identifying these “masked” PEI cases in clinical practice and monitoring their response to PERT. We observed no statistically significant difference in serum amylase and lipase levels between the study and control groups. This aligns with findings by Ventrucci et al. [20], who noted that while these enzymes rise during acute pancreatic injury, patients with severe PEI often maintain normal amylase and lipase levels. This underscores the limitation of standard serum enzyme assays in diagnosing exocrine functional capacity.

The PEI test serves as a crucial PRO tool because it directly quantifies subjective symptoms. Our methodology utilized Parts A (abdominal symptoms) and B (bowel movements) for initial diagnosis, excluding the quality-of-life questions (Part C) during the baseline assessment of treatment-naive patients. When comparing symptom prevalence, our findings were remarkably similar to those of Johnson et al. [8,15]. For instance, epigastric pain was reported by 49.7% of our patients versus 49% in Johnson’s study. However, we noted a higher prevalence of gas-related symptoms (68.1% vs. 33%) and malodorous stools (70.3% vs. 42%). Most importantly, a significant positive correlation was found between the questions in Part B (specifically fatty stools) and objective acid steatocrit values. This correlation confirms that the PEI-test is not merely a subjective survey but a reflection of actual fat malabsorption. One of the strengths of our study is the observed improvement in symptom scores following pancreatic enzyme replacement therapy as assessed by the PEI test. However, symptom improvement following pancreatic enzyme replacement therapy should not be considered diagnostic for PEI but rather supportive evidence, and a placebo effect cannot be completely excluded. Post-treatment scores showed a significant decline, indicating that the tool is sensitive to clinical changes following PERT. Since literature regarding the PEI test is currently limited largely to the pioneering work of Johnson et al., our study provides valuable independent validation of this instrument in a different geographical and clinical setting [8,15].

The findings of the present study underscore the possibility that PEI is a frequently overlooked underlying cause of dyspeptic complaints in primary clinical settings. Many patients conventionally classified under the broad umbrella of functional dyspepsia may, in fact, be suffering from undiagnosed pancreatic insufficiency, as suggested by the literature [4,21,22,23,24,25,26]. This necessitates a more vigilant diagnostic approach toward pancreatic function in cases that do not respond to standard dyspepsia treatments. The clinical utility of traditional objective markers, such as fecal elastase, is often hampered by limited access and a notable lack of sensitivity in detecting mild pancreatic dysfunction [27,28]. Consequently, validated symptom-oriented instruments offer a more pragmatic alternative, enabling clinicians to streamline diagnostic work-ups or initiate empiric PERT in appropriate candidates [4,24,26,29]. Prioritizing the early identification of PEI not only enhances symptom management but also mitigates the risk of diagnostic inertia, preventing patients from being erroneously confined to a ‘functional’ diagnosis for extended periods [19,23,24,25,30].

### 4.1. Study Limitations

While our study provides significant insights into the role of PEI in dyspepsia, several limitations must be acknowledged: The retrospective nature of the study may introduce selection bias, although we applied rigorous exclusion criteria to minimize confounding variables. We relied on the validated PEI-test and acid steatocrit rather than “gold standard” invasive tests like the secretin–cholecystokinin test or ^13^C-mixed triglyceride breath tests, which are often unavailable in routine clinical practice. The study was conducted at a single tertiary center. Larger, multi-center prospective trials are needed to confirm the generalizability of our findings across diverse populations. The lack of concurrent fecal elastase-1 measurements in all patients limits the head-to-head comparison between the PEI-test and traditional biochemical markers. Additionally, steatocrit measurements may be influenced by dietary fat intake; however, detailed dietary intake information was not available due to the retrospective design of the study. Furthermore, symptom improvement following pancreatic enzyme replacement therapy may partially reflect a placebo effect, which cannot be excluded in the absence of a placebo-controlled design.

### 4.2. Clinical Direction

Our findings suggest a paradigm shift in the management of refractory dyspepsia. Clinicians should maintain a high index of suspicion for Pancreatic Exocrine Insufficiency in patients who are traditionally diagnosed with “functional” dyspepsia, especially those presenting with malodorous stools or significant bloating. The PEI test serves as a cost-effective, non-invasive, and easily implementable screening tool that can guide the initiation of PERT. Future research should focus on establishing standardized cut-off values for the PEI-test to categorize disease severity more precisely.

### 4.3. Conclusions

This study demonstrates that a substantial proportion of patients with dyspeptic symptoms may have suspected pancreatic exocrine dysfunction that remains undetected by standard investigations. The PEI test showed an association with objective markers of fat malabsorption and may be useful in monitoring symptom changes following pancreatic enzyme replacement therapy. By integrating this symptom-based tool into routine clinical practice, healthcare providers can achieve earlier diagnosis, reduce the burden of unnecessary diagnostic procedures, and significantly improve patient outcomes through targeted enzyme therapy.

## Figures and Tables

**Table 1:** Structure and scoring of the PEI test.

Section	Number of Items		Description
Part A	7		Abdominal symptoms
Part B	6		Bowel movement-related symptoms
Part C	5		Impact on quality of life
Scoring method			
**Patient group**		**Formula**	
Newly diagnosed patients		(Part A + Part B)/2	
Follow-up patients		(Part A + Part B + Part C)/3	
Severity classification			
**Score**		**Interpretation**	
0.6–1.4		Mild PEI	
1.4–1.8		Moderate PEI	
≥1.8		Severe PEI	

**Table 2:** Comparison of Demographic Data Between Study and Control Groups.

	PEI (*n* = 91)	Control (*n* = 58)	*p*
Amylase (U/L)			*p* = 0.297
Mean ± SD	68.47 ± 24.22	64.96 ± 22.33	
Min–max	20–159	35–123	
Lipase (U/L)			*p* = 0.814
Mean ± SD	32.94 ± 17.52	30.50 ± 17.80	
Min–max	14–140	8–90	
Steatocrit			*p* < 0.001
Mean ± SD	1.71 ± 1.05	1.95 ± 0.53	
Min–max	1–1.79	1.71–3.66	
Vitamin D (ng/mL)			*p* = 0.722
Mean ± SD	15.40 ± 7.66	16.74 ± 12.97	
Min–max	4.74–36	3.12–98	
HbA1c (%)			*p* = 0.195
Mean ± SD	6.1 ± 1.21	5.81 ± 0.78	
Min–max	5.10–13	4.9–9.4	
Folic acid (ng/mL)			*p* = 0.289
Mean ± SD	7.17 ± 2.59	7.91 ± 3.13	
Min–max	2.40–14	3.60–18	
B_12_ (pg/mL)			*p* = 0.668
Mean ± SD	372.27 ± 232.81	361.48 ± 255	
Min–max	1.96–2000	144–1163	

## Data Availability

The data supporting the findings of this study are available from the corresponding author upon reasonable request.
